# Action, emotion, and music-colour synaesthesia: an examination of sensorimotor and emotional responses in synaesthetes and non-synaesthetes

**DOI:** 10.1007/s00426-023-01856-2

**Published:** 2023-07-15

**Authors:** Caroline Curwen, Renee Timmers, Andrea Schiavio

**Affiliations:** 1 Department of Music, The University of Sheffield, Jessop Building, 34 Leavygreave Road, Sheffield, S3 7RD UK; 2 School of Arts and Creative Technologies, University of York, Sally Baldwin Building D, York, YO10 5DD UK

## Abstract

Synaesthesia has been conceptualised as a joining of sensory experiences. Taking a holistic, embodied perspective, we investigate in this paper the role of action and emotion, testing hypotheses related to (1) changes to action-related qualities of a musical stimulus affect the resulting synaesthetic experience; (2) a comparable relationship exists between music, sensorimotor and emotional responses in synaesthetes and the general population; and (3) sensorimotor responses are more strongly associated with synaesthesia than emotion. 29 synaesthetes and 33 non-synaesthetes listened to 12 musical excerpts performed on a musical instrument they had first-hand experience playing, an instrument never played before, and a deadpan performance generated by notation software, i.e., a performance without expression. They evaluated the intensity of their experience of the music using a list of dimensions that relate to sensorimotor, emotional or synaesthetic sensations. Results demonstrated that the intensity of listeners’ responses was most strongly influenced by whether or not music is performed by a human, more so than familiarity with a particular instrument. Furthermore, our findings reveal a shared relationship between emotional and sensorimotor responses among both synaesthetes and non-synaesthetes. Yet it was sensorimotor intensity that was shown to be fundamentally associated with the intensity of the synaesthetic response. Overall, the research argues for, and gives first evidence of a key role of action in shaping the experiences of music-colour synaesthesia.

**Supplementary Information:**

The online version contains supplementary material available at 10.1007/s00426-023-01856-2.

## Introduction

Synaesthesia has traditionally been described in neurology as the merging of the senses, emphasising cross-sensory co-activation upon uni-sensory perception, emphasising direct connectivity between sound and colour in the case of music (Cytowic, 2002, p. 325). Acknowledging the central role of action for perception and cognition, we recently challenged this perspective putting the potential role of sensorimotor experiences in music-colour synaesthesia to the fore (Curwen, 2020, 2022a). Music is known to present rich cross-modal sensorimotor associations including with action, and various sensory dimensions (Eitan & Timmers, 2010; Reybrouck, 2017). This paper investigates the relevance of such associations for music-colour synaesthesia presenting hypotheses for sensory and action-related phenomena as synaesthetic inducers. It systematically compares synaesthetic responses to stimuli performed on a musical instrument participants had first-hand experience playing, an instrument never played before and an electronic deadpan performance generated by notation software, i.e., a performance without expression. The intention behind the creation of the deadpan stimuli was to achieve a non-human-like performance, minimising expression, sensorimotor simulation, and embodiment in the listening experience. Furthermore, the study compares relationships between the following different types of responses: synaesthetic, emotional and sensorimotor associations, where the latter refers to common multimodal associations grounded in active engagement with the environment (Barsalou, 1999, 2003; Myin, 2016). It tests synaesthetes and a control group to explore parallels between processing of music in controls and synaesthetes. Whilst previous research has examined the mediating role of emotion in music-colour synaesthesia (Isbilen & Krumhansl, 2016; Lebeau & Richer, 2022; Palmer, et al., 2013, 2016; Ward, 2004) little is known about the role of performance-related actions, nor about the relative strength of the connection with emotion compared to sensorimotor associations.

### Music-colour synaesthesia

Music-colour synaesthesia is included under the umbrella term “coloured hearing”, or “chromesthesia” (Ward et al., 2006a, b). The four broad categories of musical inducer^1^ include compositional style, timbre, tonality, and pitch (tone) (Peacock, 1985). Synaesthetic experiences induced from musical stimuli commonly include colour but can also extend to visual-spatial features such as shapes, spatial layouts, and textures (Eagleman & Goodale, 2009). Due to the idiosyncratic nature of their experiences, synaesthetes frequently exhibit variations in the colours and imagery associated with specific musical inducers. Additionally, each synaesthete may possess a distinct combination of various types of synaesthetic inducer (Curwen, 2018; Mills et al., 2003).

Although synaesthesia is often described as being cross-sensory or “a union of the senses'' (Cytowic, 2002, p. 325), it has also been shown that in some forms of the condition activation can be triggered by concepts (van Leeuwen et al., 2015). This has led to the development of alternative theories such as *ideaesthesia* (Jürgens & Nikolić, 2012; Nikolić, 2009) and a replacement definition: “Synaesthesia is a phenomenon in which a mental activation of a certain concept or idea is associated consistently with a certain perception-like experience” (Nikolić, 2009, p. 28). Indeed, the synaesthetic experience of music is not just about the experience of associating a colour with individual tones or chords, nor is it solely about sound properties as inducers (Mills et al., 2003). The experience is mediated by factors such as emotion and tempo and, for some, a synaesthetic response can only be elicited in the context of listening to a substantial passage of music (Curwen, 2018). For example, the strength of synaesthetic experience is likely to be greater for music that elicits a strong emotional response than from one that does not (Marks, 2004). Gathering support for this hypothesis, Isbilen and Krumhansl (2016) tested musicians, non-musicians, absolute pitch possessors, and music-colour synaesthetes. Their results indicate similarities between participants’ colour and emotional associations with music and their matching between colours and emotions, suggesting a mediating role for emotion in colour assignment to music. Further similarities between the experiences of synaesthetes and non-synaesthetes can be observed through common cross-modal associations made in the general population. For example, pitch height, lightness, size, and brightness are frequently correlated and strongly associated in perception (Eitan & Timmers, 2010; Marks, 1974, 1987; von Hornbostel, 1925; Ward et al., 2006a, b). Such parallels between synaesthetes and non-synaesthetes (Simner, 2012) suggest that certain similarities and differences may be a matter of degree, suggesting a continuum in underlying processes, at least for some forms of synaesthesia.

### An “act of doing”

Accounts of perception in typical music cognition have embraced embodied and enactive frameworks describing engagement with music from a sensorimotor perspective i.e., as an “act of doing”. Such views highlight the importance of kinesthetic experience and bodily based engagement with a material context for music cognition (Krueger, 2009, 2011, 2014; Loaiza, 2016; Maes et al., 2014; Reybrouck, 2014, 2017; Schiavio et al., 2017, 2019; van der Schyff et al., 2022). Scholars working from an embodied perspective argue that mental life is not well captured by analyses of mental states (i.e., their functional laws or computational underpinnings), nor by singular study of the brain’s structural and functional properties. Rather, understanding of mind and subjectivity requires integration with an analysis of situated bodily action and its experience (Gallagher, 2006; Thompson, 2007). This offers the opportunity to explore how brain-body-world synergies form and operate via a continuous integration of exogenous and endogenous factors, helping us look at how we think, act, and communicate with others from a rich orientation, offering insights that might remain obscured in more traditional accounts of the mental (see Johnson, 2007; Varela et al., 1991). In music scholarship, these ideas have been variously defended and developed (van der Schyff et al., 2022), presenting new possibilities to understand musical activities such as composition (Nagy, 2016), learning (Bowman, 2004), and listening (Kozak, 2020; Leman & Maes, 2014) following Clarke’s (2005) remarks:

The intimate relationship between perception and action in the ways that players listen takes many forms, depending—among other factors… on the specific physical characteristics of the instruments in relation to the human body… (p. 152).

The close relationship between music perception and action is further evidenced in neuroimaging research. Margulis et al. (2009) found that expert flautists showed more motor activity when listening to Bach’s Partitas for solo flute than when they were listening to Bach’s Partitas for solo violin. The same was true for expert violinists listening to the flute Partitas. Neither group was asked to engage in any motor activity or to play their instrument, but to only passively listen, illustrating the “special experience instrumentalists have with music for their instrument of expertise” (p. 271). This arguably refers to the notion of embodied simulation (Gallese, 2005) indicating a mechanism that allows an agent to use its cognitive system to internally replicate (i.e., “simulate”) another’s actions and feelings (Gallese, 2014). The motor knowledge of competent listeners may automatically activate the same motor program required to reproduce the music while listening (Overy & Molnar-Szakacs, 2009). When there is little match between the actions “implied” in the auditory signal and the instrumental expertise of the listener (i.e., when a violinist is listening to a familiar piece performed on a piano), less motor activity in the brain has been reported, reflecting the tight association of action and perception at a neural level. Influences of instrumental expertise on cross-modal correspondences have also been shown when comparing flautists and pianists and their mapping of pitch in horizontal space (Timmers & Li, 2016). It is proposed here that such embodied motor knowledge is likely to extend to forms of music-colour synaesthesia which may share a similar grounding in action to typical music cognition (Curwen, 2020) leading to the following predictions that are examined in this paper.

We predict a difference in strength of synaesthetic experience depending on a listener’s motor associations with an instrument: whether or not the listener is proficient in playing the instrument; or whether it is an electronic deadpan performance. We also predict synaesthetic experience to have a strong relationship with induced drives for action (such as the desire to sing/hum or move/dance along) and cross-modal sensorimotor experiences. We interpret the cross-modal dimensions as sensorimotor responses, following demonstrations that many cross-sensory correspondences have a grounding in action (Spence, 2011): touching, lifting, moving as well as producing sounds using instruments with a range of properties (Colombetti & Roberts, 2015; Reybrouck, 2017; Stewart et al., 2003). These associations are based on the individual's past experiences of interacting with the world through their body movements, as well as the interaction between different sensory modalities (Barsalou, 1999, 2003; Myin, 2016). For example, Curwen (2022a) investigated synaesthesia for written musical key signatures and proposed that written notation on the stave carried sensorimotor information about the hand shape employed in sound production. We allude more generally to cross-modal correspondences with sounds, e.g., associations between musical sound properties and physical properties including size, shape, and energy for which the act of sound production seems an important underlying factor (Eitan & Timmers, 2010).

### Hypothesis and aims

The association between emotion, music, and colour demonstrated in both synaesthetes and non-synaesthetes (Palmer et al., 2013, 2016) suggests that emotions serve as a primary mediator in typical music-colour associations. Consequently, previous research has primarily focused on the mediating role of emotion in synaesthesia associated with music (Isbilen & Krumhansl, 2016; Lebeau & Richer, 2022; Palmer et al., 2013, 2016; Ward, 2004) and has paid little attention to the influence of performance-related actions and sensorimotor associations.

An empirical study was designed to examine the interplay between emotion, action, and sensorimotor experiences as a mediator of synaesthesia and to test the following hypotheses: Changes to action-related qualities of a musical stimulus affect the resulting synaesthetic experience, predicting that (H1) the intensity of a listener’s synaesthetic experience will be influenced by a change of musical instrument (i.e., a change from their own instrument, to one with which they have no expertise); (H2) the intensity of a listener’s synaesthetic experience will be affected by the performance (i.e., whether or not the instrument is played by a human).

Synaesthetes and controls will show similar relationships between music, sensorimotor and emotional responses with sensorimotor responses taking prominence, predicting the following: (H3) synaesthetes and non-synaesthetes will rate emotional and sensorimotor factors similarly across different listening conditions; (H4) emotional responses will feed into synaesthetic responses; (H5) sensorimotor responses will feed into synaesthetic responses, and more strongly so than emotion.

In summary, the main aim of this study was to investigate potential commonalities between the mechanisms underlying music-colour synaesthesia and typical music cognition, and to test the key role of role of action in shaping the experiences of music-colour synaesthesia.

## Method

### Participants

Participants were recruited through voluntary sign-up via social media, a volunteers list, and the first author’s own contacts. Synaesthetes were also recruited from the *UK Synaesthesia Association* and the *International Association of Synaesthetes, Artists and Scientists.* The main inclusion criteria were to be an active performing musician (amateur, student or professional) and to be open to listening to performances of the musical material on different instruments (see “Procedure”). A total of 62 volunteers took part in the study: 29 music-colour synaesthetes and 33 controls. Participants in both groups had a spread in age, gender and musical training. Musical ability was high with the majority of participants indicating proficiency levels of ABRSM Grade 8 or higher (see Table 1).

**Table 1:** Summary of participant characteristics: gender, age, absolute pitch, and level of musicianship

Characteristic	Synaesthetes	Controls
Male	16	12
Female	13	21
Age	18–65	16–67
AP	7	4
Professional	11	15
Very high level amateur	7	7
Grade 8	6	8
Intermediate	3	3
Beginner	2	–

*AP* absolute pitch, *Grade 8* Grade 8 of the Associated Board of the Royal Schools of Music

Music-colour synaesthesia was described to participants as spontaneously seeing colours and shapes on hearing music. Those who self-identified as a music-colour synaesthete were invited to describe their response to a piece of music that elicits a very strong synaesthetic experience and one that does not, and to provide a brief description of why. Reported musical inducers were varied and not confined to the more commonly examined tone-colour synaesthesia. For this reason, the identification of synaesthetes using a battery such as The Synaesthesia Battery (“The Battery''—see Eagleman et al., 2007) was considered inappropriate. The Battery has been used successfully to verify the presence of some forms of synaesthesia, but it has limitations when testing for music-colour synaesthesia. Although the Battery is able to test for single tones to colours, single chords to colour, and instrumental timbre to colour (Zamm et al., 2013), music-colour synaesthesia is reported to be a far richer phenomenon. Often synaesthetes require an extract or an entire piece of music to elicit a response, which may then be further mediated by tempo, emotion and timbre. Moving maps, shapes, landscapes and textures may also accompany the colours. The experience is not well represented by the ability to ‘colour-label’ tones and chords in isolation (Curwen, 2018; Marks, 2011; Mills et al., 2003). To avoid overlooking certain forms of music-colour synaesthesia, self-reporting synaesthetes in this study were asked to provide examples of their synaesthetic experience. These were screened as part of the inclusion selection for the synaesthete group (see Appendix E for examples). No-one was excluded following the exercise, but it was clear that synaesthetic experiences for music were varied and idiosyncratic. A number of factors were reported to include synaesthesia including orchestral timbre, harmonic textures, tonality, perceived movement, and level of emotional engagement. Concurrents were reported to manifest as colours, monochrome shades, shapes, moving landscapes, and sensations of texture or taste. Not all synaesthetes experienced the same phenomena, or in the same combination.

### Design

Two independent measures were controlled in a factorial design as follows: a between-participant variable of group (Synaesthetes vs. Controls) and a within-participant variable related to the performance of the music on a principal or unknown instrument or electronically generated. Three dependent variables were considered grouping ratings related to synaesthesia, emotional and sensorimotor associations.

### Materials

Four musical excerpts were used as musical material, and two sets of questionnaire material were used for the two phases of the study. Phase 1 collected demographic information for pre-screening of participants including whether participants experienced synaesthesia for music (see Appendix A). Phase 2 collected the core data of the empirical study, including self-reported responses to presented musical excerpts, questions about perceived emotion and perceived sensorimotor associations, and for the synaesthetes perceived synaesthetic responses (see Appendix B). Data was collected using an online platform.

Musical material consisted of four contrasting dances^2^ (Prelude, Courante, Sarabande and Gigue) from J.S. Bach’s Cello Suite No.1 BWV 1007 in G Major. These were presented on three instruments totalling twelve musical excerpts:

- Set 1: four excerpts (one from each dance) performed on the participant’s Principal Instrument
- Set 2: as in Set 1 performed on an Unknown Instrument
- Set 3: as in Set 1 produced on an Electronic Instrument

J.S. Bach’s Cello Suite No.1 has been transcribed for a variety of instruments which allowed bespoke performances of the excerpts to be created for each participant: comprising a performance on a familiar instrument, and an unfamiliar instrument. Professional recordings, readily available from Spotify and YouTube, were converted to MP3 files and trimmed to 30–60 s in length. This was to ensure that the excerpt ran from the beginning of the dance but always finished at the end of a phrase, and at a point that made musical sense. A list of all the recordings used are detailed in Appendix C. MP3 files of the Electronic version of the excerpts were created using the notation software package, MuseScore 3.5. Pitches and note values were entered manually into the software without dynamics, phrasing, articulation or expression, resulting in a flat, mechanical rendition (i.e., deadpan).

### Procedure

Ethical approval was obtained from the departmental research ethics committee at the University of Sheffield, and all participants gave informed consent at the start of the online survey, followed by Phase 1 of the study collecting demographic information and details related to their level of musicianship (see Appendix D), their principal instrument, and whether they had synaesthesia for music.

A pre-trial of the main section of the study presented an excerpt of the Bach Cello Suite performed on Marimba. Because Bach’s unaccompanied Cello Suites occupy a profound place in Western Art-music canon, this was used to assess whether participants were sufficiently accepting towards this music performed on an instrument other than a cello before they continued with the main part of the study (Phase 2).

Phase 2 of the experiment was tailored to participants’ instrumental choices. Participants were invited to select the principal instrument they play and another instrument that they had never played before from a preset list of instrumental pairings. Those whose familiar and unfamiliar instruments did not fit the instrument pairing selections presented, could request bespoke musical materials for Phase 2. Participants used headphones to listen to the twelve excerpts presented in pseudo-randomized order: no version of the same excerpt was directly preceded or followed by another version. After each excerpt, participants evaluated the applicability of a term (see Table 2) to describe their experience of the music. Terms used to evaluate musical excerpts comprised seven pairs of emotional dimensions (Cowen et al., 2020), eight pairs of cross-modal sensorimotor dimensions (Eitan & Timmers, 2010), five synaesthetic experience questions derived from observations by Eagleman and Goodale (2009), and four drive towards action questions (Janata et al., 2011)—see Table 2. A five-point scale was used for unidirectional scales, representing 1 (*not at all*), 2 (*weakly*), 3 (*moderately*), 4 (*strongly*) and 5 (*very strongly*) (see Appendix D). Bidirectional scales were employed using a nine-point scale, with the midpoint identified. This meant that it was not possible to rate both poles. For example, when considering rough/smooth, participants could only rate rough or smooth, or indicate that neither term was applicable.

**Table 2:** Overview of terms used to evaluate each musical excerpt assessing emotional, sensorimotor, drive towards action and synaesthetic responses

Emotional	Sensorimotor	Drive towards action	Synaesthetic
Positive|Negative	Rough|Smooth	Actually sing/hum along?	Dark/Light shades
Excited|Calm	Light|Heavy	Imagined sing/hum along?	Textures
Weak|Strong	Spiky|Round	Actually move/dance along?	Movement
Tense|Relaxed	Bright|Dark	Imagined move/dance along?	Colour
Happy|Sad	Dynamic|Static		Shapes
Angry|Tender	Thick|Thin		
Pleasant/Unpleasant	Warm/Cold		
	Spatially High/ Spatially Low		

The synaesthete group also rated strength of their synaesthetic experience in terms of the presence of dark or light shades, colour, texture, shapes and movement (Eagleman & Goodale, 2009, p. 4). No time limit was applied and participants were permitted to play the excerpt multiple times. The experiment (Phase 2) lasted around 30 min.

### Data processing and analysis

All ratings were transformed into unipolar ratings from 1 (*not at all*) to 5 (*very strongly*) so we could calculate the strength of experience per type of dimension (emotional, sensorimotor, synaesthetic) irrespective of the individual dimension most strongly perceived to be applicable. Drive towards action ratings were not analysed separately but were included within the sensorimotor dimension. As data approximated an interval scale measuring strength of response per participant, these Likert scale responses were summed and a mean score calculated for the strength of three ratings in the synaesthete group (sensorimotor, emotional, and strength of synaesthetic response) and two ratings in the control group (sensorimotor and emotional).

The data analysis is split into two parts—the first looks at the data that is shared between the controls and synaesthetes and analyses emotional and sensorimotor ratings by these groups. The second examines the responses of the synaesthetes only, including all types of responses as follows: emotion, sensorimotor, and synaesthesia.

## Results I: synaesthetes and controls

Emotional and sensorimotor responses in synaesthetes and controls were subjected to three types of analyses as follows: First, we examined the effect of Instrument (Principal, Unknown, or Electronic) on the mean strength of emotional and sensorimotor ratings. This analysis examined whether a change from an instrument the participants were very familiar with to one they had no experience playing would significantly diminish the strength of ratings, and whether it mattered if music was performed or computer generated in a dead-pan manner. Second, we examined the relationship between emotional and sensorimotor dimensions using a Principal Component Analysis (PCA) and tested differences between the two participant groups in PCA scores. Last, the strength of the relationship between sensorimotor and emotional responses was examined for each group separately in a correlation analysis. An observable relationship between emotional and sensorimotor response was expected in both groups, and no significant differences between controls and synaesthetes.

### Descriptive statistics

**Fig. 1:**
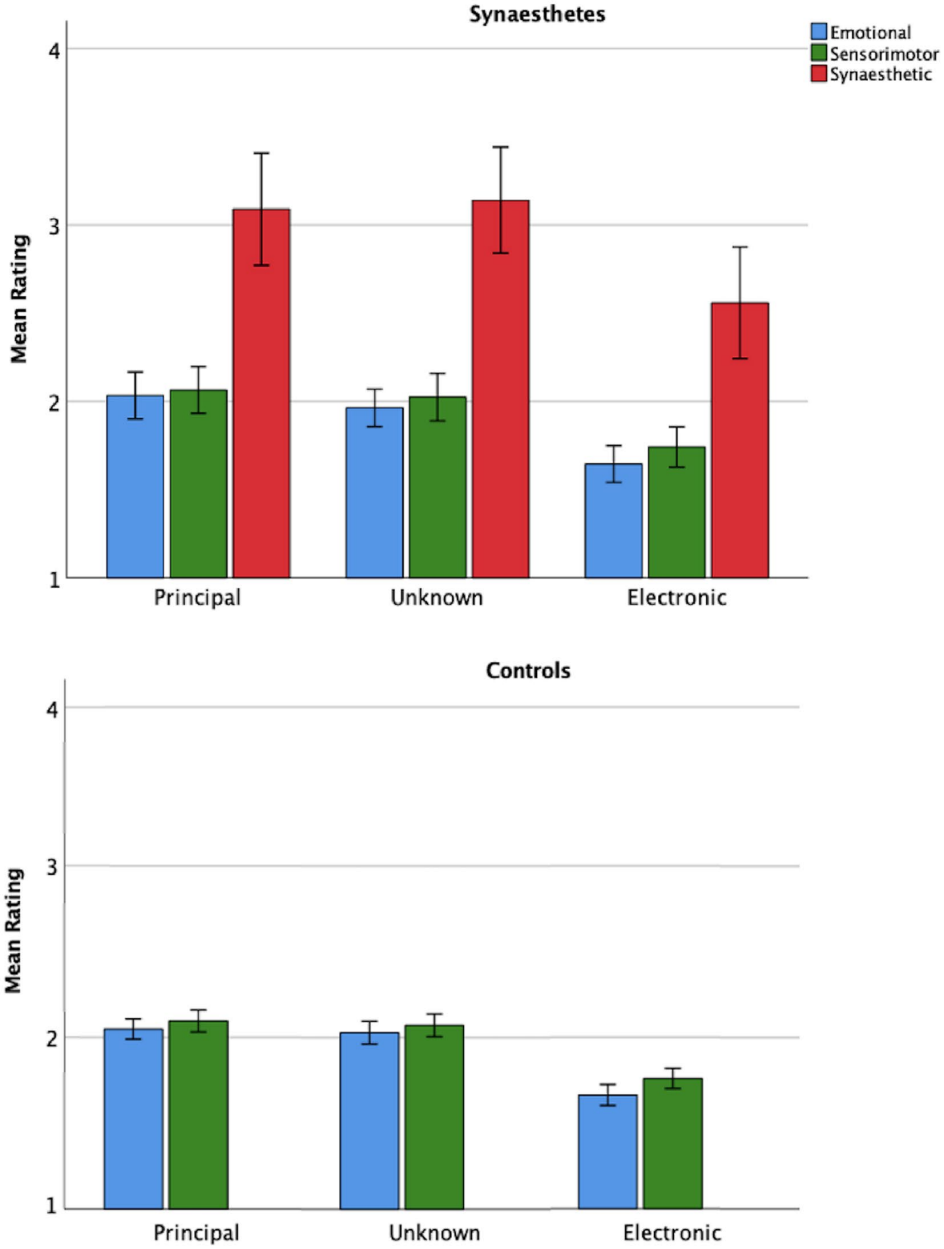
Mean synaesthetic, emotional, sensorimotor ratings in the synaesthete group and control groups by instrument. *Principal* principal instrument, *Unknown* unknown instrument, *Electronic* electronic instrument, *Error Bars* + / − 2 standard errors

Mean emotional and sensorimotor ratings in both groups shared similar patterns and were weaker in the Electronic condition compared to Principal and Unknown conditions (Fig. 1). Generally, the Principal condition exhibited similar or higher ratings for emotional and sensorimotor responses compared to the Unknown condition. Overall, the similarity in emotional and sensorimotor ratings in both groups was as expected.

### Effect of group and instrument on responses

A 2 × 3 mixed measures ANOVA was conducted for each type of rating (emotion and sensorimotor) with music-colour synaesthesia (Yes, No) as a between-subjects factor, and instrumental conditions (Principal, Unknown, Electronic) as within-subjects factors. SPSS Statistics V27 was used to analyse the data.

No main effects of the participant group (presence of synaesthesia) were observed, and no interactions with synaesthesia, confirming emotional and sensorimotor ratings in both groups varied similarly across conditions (see Table 3 and Fig. 1). Significant differences concerned effects of Instrument for both dependent variables further analysed next.

**Table 3:** Results for strength of sensorimotor and emotional response in a two-way 2 × 3 mixed measures ANOVA

Variable	Sensorimotor response				Emotional response			
	*df*	*F*	*η*^*2*^	*p*	*df*	*F*	*η*^*2*^	*p*
Syn	(1, 60)	.183	.003	.670	(1, 60)	.480	.008	.491
Syn*Instr	(2, 120)	.076	.001	.927	(2, 120)	.202	.003	.818
Instr	(2, 120)	31.230	**.342**	.001	(2, 120)	57.060	**.487**	.001

*Syn* synaesthesia, *Instr* instrument, *df* degrees of freedom, *F* F ratio, *η*^***2***^ effect size

Larger effect sizes are bolded

Post hoc Bonferroni tests indicated the Electronic condition (*M* = 1.66, *SD* = .41) had significantly (*p* =  < .001) lower emotional ratings than the Principal condition (*M* = 2.03, *SD* = .44) and Unknown condition (*M* = 2.0, *SD* = .42). It showed no significant differences between the Principal and Unknown condition (*p* = 1).

Post hoc Bonferroni tests indicated the Electronic condition (*M* = 1.76, *SD* = .42) had significantly (*p* =  < .001) lower sensorimotor ratings than the Principal condition (*M* = 2.08, *SD* = .46) and the Unknown condition (*M* = 2.05, *SD* = .47). It showed no significant differences between the Principal and Unknown condition (*p* = 1).

In summary, the cause of the main effect of Instrument type on sensorimotor and emotional ratings was the performance played without expression in the Electronic condition.

### Clustering of individual dimensions (PCA)

The previous analysis examines emotional and sensorimotor responses separately. A Principal Component Analysis (PCA) was conducted on individual dimensions to examine the interrelation, or clustering, of sensorimotor and emotional dimensions. The suitability of data for PCA was assessed prior to analysis. The correlation matrix showed all but one variable had at least one correlation coefficient greater than .3. This variable (Actually Singing Along) was removed from the analysis. Kaiser–Meyer–Olkin (KMO) measures for individual variables were inspected and nine variables measuring less than 0.5 (Static, Angry, Weak, Thick, Thin, Tender, Imagine Singing Along, Imagine Moving, Actually Moving) were also removed from the analysis. The overall KMO measure was 0.72 and individual KMO measures were all greater than 0.6: classifications of “mediocre” to “middling” according to Kaiser (1974). Bartlett's test of sphericity was significant (*p* = .001), indicating the data was likely to be factorisable.

PCA revealed five components with eigenvalues > 1 explaining 26.5%, 18.1%, 13.23%, 8.44% and 5.89% of the total variance, respectively. However, a four-component solution met the interpretability criterion and this solution explained 66.27% of the total variance aided by a “simple structure” (Thurstone, 1947) and Varimax orthogonal rotation. Component 1 (positive emotion) indexed positive valence and to a lesser degree high arousal, and had strong loadings on *positive, pleasant*, *happy* and *warm*. It loaded negatively with negative valence and low arousal *sadness*. Component 2 (relaxed/smooth) had high loadings on a mixture of emotional and sensorimotor items indexing low arousal, positive valence, and smoothness: *relaxed*, *smooth*, *calm* and *round*. Component 3 (rough/tense) had high loadings primarily on sensorimotor items, but with emotional connotations of negative valence and high arousal: *rough, tense, spiky*, and *cold*. Component 4 (light/bright) related strongly to sensorimotor characteristics, but not with emotional characteristics. These are commonly observed cross-modal correspondences indexing pitch height with brightness and lightness (Gallace & Spence, 2006; Marks, 1987, 2004; Mondloch & Maurer, 2004). *Heavy* and *dark* indicated the reverse and were negatively loaded together with *spatially low* with a negative cross-loading of over 0.4. Component loadings and communalities are presented in Table 4. These results show distinctions and associations between the two types of evaluations.

**Table 4:** Rotated structure matrix for PCA with varimax rotation for a four component solution for sensorimotor and emotional ratings in synaesthetes and controls

Rotated components coefficients					
Item	26.5%	18.1%	13.23%	8.44%	Communalities
	Component 1	Component 2	Component 3	Component 4	
Positive	**.896**	.171	.009	.170	.665
Pleasant	**.846**	.175	− .084	.197	.552
Happy	**.845**	.142	− .011	.284	.479
Warm	**.840**	.150	− .101	− .018	.730
Dynamic	**.716**	− .038	.231	.137	.663
Excited	**.685**	− .096	.473	− .029	.615
Unpleasant	** − .551**	.374	.414	.026	.862
Negative	** − .544**	.449	.387	− .133	.704
Sad	** − .494**	.472	.308	− .318	.814
Relaxed	.147	**.791**	− .281	.058	.793
Smooth	.297	**.749**	− .167	.084	.685
Calm	− .092	**.717**	− .157	− .073	.496
Round	.349	**.573**	− .143	− .158	.675
Spatially Low	− .029	**.553**	.124	** − .467**	.672
Strong	.433	**.435**	− .001	− .321	.540
Rough	− .026	− .163	**.792**	− .056	.658
Tense	.155	− .178	**.780**	− .094	.754
Spiky	− .019	− .138	**.744**	.135	.591
Cold	− .310	.505	**.567**	.017	.786
Light	.296	.198	.142	**.779**	.586
Heavy	.163	.308	.200	** − .761**	.739
Bright	.520	.057	.076	**.711**	.433
Dark	− .102	**.467**	.189	** − .641**	.673
Spatially High	.256	− .009	.345	**.498**	.740

Major loadings for each item are bolded

Subsequently, independent samples *t*-tests were used to determine whether synaesthetes and controls scored differently on Components 1, 3 and 4. Scores were normally distributed, as assessed by Shapiro–Wilk’s test (*p* > .05). A Mann–Whitney test was run for Component 2 data, as scores were not normally distributed. No significant differences between scores of the two groups were observed for any of the Components (*p* > .28).

### Relationship between emotional and sensorimotor ratings per group

**Fig. 2:**
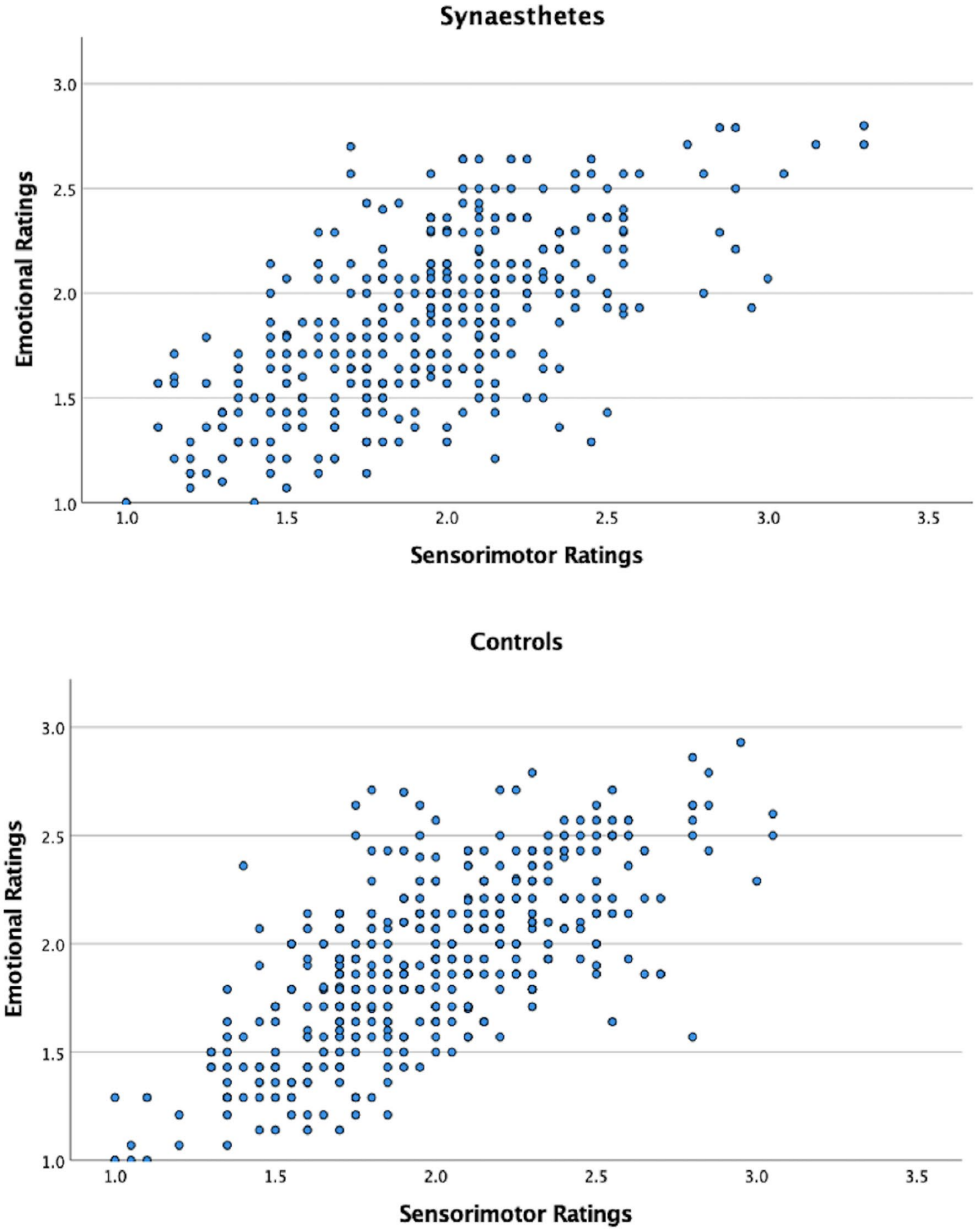
Scatter plots showing associations between sensorimotor and emotional ratings for synaesthetes (top) and controls (bottom)

Finally, a correlation analysis tested the relationship between sensorimotor and emotional responses to music. This was done separately for the two groups. There was a significant correlation between sensorimotor and emotional responses in both the control group; *r* (394) = .71, *p* =  < .001) and the synaesthete group; *r* (346) = .64, *p* =  < .001 as expected. The correlations are illustrated in Fig. 2. This shows a similar trend in both groups, but more variability or idiosyncrasy in the synaesthete group.

## Results II: synaesthetes

This section reports the results for the Synaesthete group, including ratings of emotional, sensorimotor and synaesthetic responses.

### Descriptive statistics

Figure 1 shows the mean strength of synaesthetic response, emotional ratings, and sensorimotor ratings in each Instrumental condition. Visual inspection indicates that the pattern of mean synaesthetic response ratings exhibits subtle differences from that of the emotional and sensorimotor ratings. Although the Electronic condition still displays the lowest ratings overall, the prediction (H1) that synaesthetes would report the highest level of intensity for synaesthetic experience in the Principal condition is not supported.

### The effects of instrument and dance on response strength

Three two-way 3 × 4 repeated measures ANOVAs analysed mean strength of synaesthetic, emotional and sensorimotor responses with Instrument (Principal, Unknown, Electronic) as within-subjects factor. There were no outliers and data were normally distributed as assessed by box plot and a Kolmogorov–Smirnov test (*p* > .05). Table 5 shows the results of the ANOVAs. The main effect of Instrument was significant for synaesthetic responses, emotional ratings and sensorimotor ratings for which post hoc Bonferroni tests were performed.

**Table 5:** Results for the strength of synaesthetic response, emotional ratings and sensorimotor ratings in three repeated measures ANOVAs

Condition	*df*	Synaesthetic			Emotional			Sensorimotor		
		*F*	*η*^*2*^	*p*	*F*	*η*^*2*^	*p*	*F*	*η*^*2*^	*p*
Instr	(2, 56)	14.92	.35	.001	19.16	.41	.001	11.99	.30	.001

*Instr* instrument, *df* degrees of freedom, *F* F ratio, *η*^***2***^ effect size, *p *significance

Post hoc Bonferroni tests indicated that the Electronic condition had significantly weaker synaesthetic responses, sensorimotor ratings, and emotional ratings than the Principal condition and the Unknown condition (*p* < .002 for all comparisons). The difference between the Principal and Unknown conditions was insignificant (*p* = 1.0).

### Relationship between types of response

**Fig. 3:**
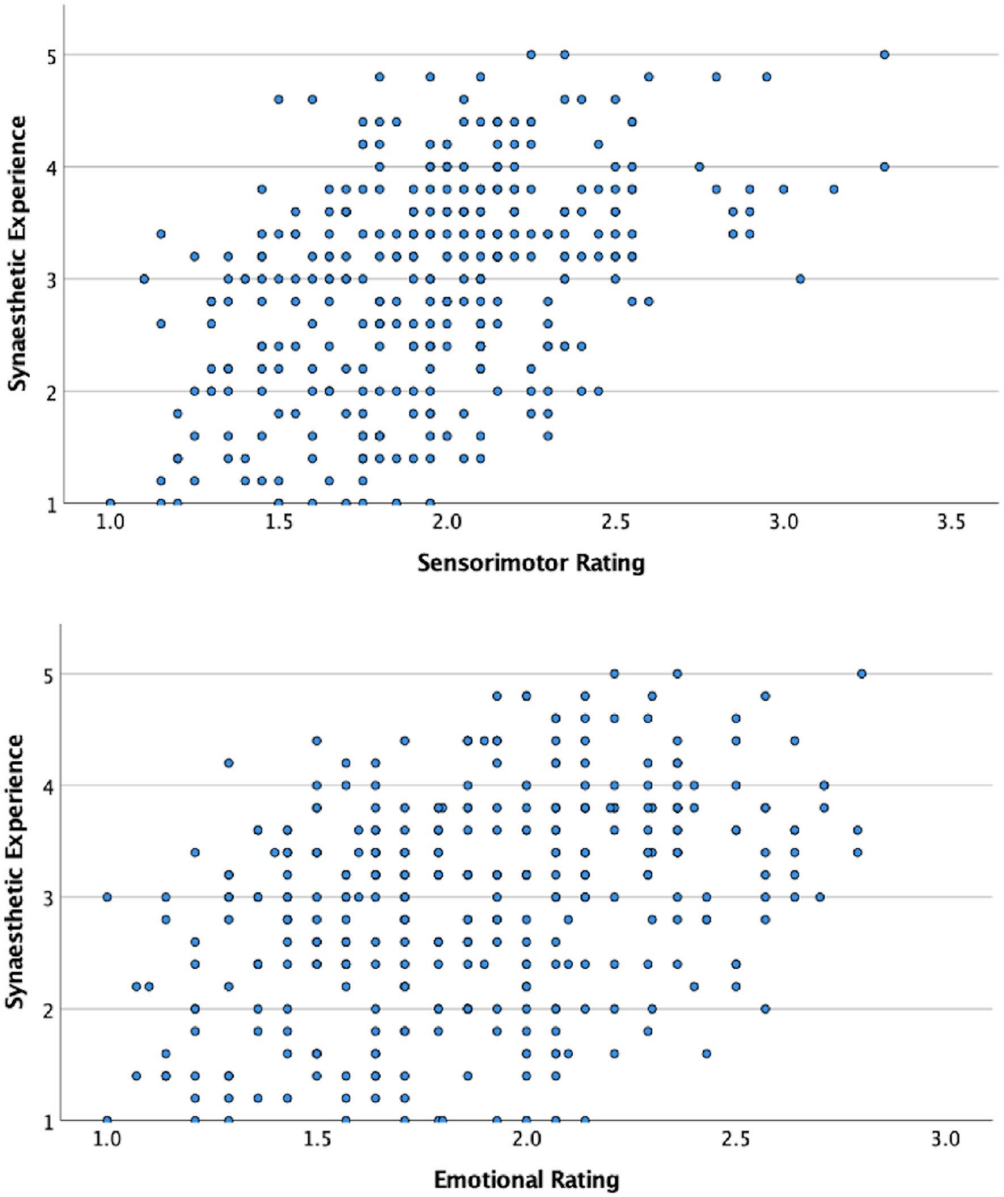
Scatter plots of synaesthetic by sensorimotor ratings (top) and synaesthetic by emotional ratings (bottom)

A correlation analysis was performed to assess the association between emotional and synaesthetic responses, and between sensorimotor and synaesthetic responses. The graphs in Fig. 3 illustrate that emotional and sensorimotor ratings are individually correlated to the strength of synaesthetic response. Yet the difference in variability between the two dimensions is also highlighted. Sensorimotor ratings and synaesthetic strength show a stronger relationship compared to emotional ratings and synaesthetic strength as reflected in a narrower distribution of data points. Correlations between sensorimotor and synaesthetic ratings; *r* (346) = .46, *p* =  < .001) and emotional and synaesthetic ratings; *r* (346) = .37, *p* =  < .001) were significant, indicating that both associations are unlikely to have arisen by chance.

This indicates a linear relationship between both pairs of responses but does not take into account overlapping variance between sensorimotor and emotional ratings. A further analysis was, therefore, conducted using Partial Correlations to assess the unique correlations between sensorimotor ratings and synaesthetic experience, and emotional ratings and synaesthetic experience, correcting for correlations with the other variable.

### Relationship between sensorimotor ratings and synaesthetic experience

Pearson's partial correlations showed the strength of the relationship between sensorimotor and synaesthetic responses was less when controlling for correlations with emotion, but remained significant (see Table 6). This indicates that the synaesthetic responses had a unique sensorimotor grounding irrespective of emotion.

**Table 6:** Correlations between the strength of sensorimotor ratings and synaesthetic response before and after controlling for emotional responses

Condition	Sensorimotor and synaesthesia		Controlling for emotion	
	Zero order	*R*^2^	First order PC	*R*^2^
Principal	*r*(114) = .463, *p* < .001	21.44	*r*(113) = .346, *p* < .001	11.97
Unknown	*r*(114) = .443, *p* < .001	19.62	*r*(113) = .395, *p* < .001	15.60
Electronic	*r*(114) = .320, *p* < .001	10.24	*r*(113) = .193, *p* < .039	3.72

*PC* partial correlation

### Relationship between emotional ratings and synaesthetic experience

Pearson’s partial correlations further showed a reduced strength of the linear relationship between emotional and synaesthetic responses when controlling for correlations with sensorimotor responses, becoming insignificant in all but the Electronic condition. This suggests a non-unique or weak relationship between the strength of synaesthetic response and emotional ratings.

These results indicate a stronger relationship between sensorimotor responses and synaesthetic responses than emotional and synaesthetic responses (Table 7).

**Table 7:** Correlations between strength of emotional ratings and synaesthetic response before and after controlling for sensorimotor responses

Condition	Emotion and synaesthesia		Controlling for sensorimotor	
	Zero order	*R*^2^	First order PC	*R*^2^
Principal	*r*(114) = .544, *p* < .001	29.59	*r*(113) = .056, *p* < .549	0.31
Unknown	*r*(114) = .227, *p* < .001	5.15	*r*(113) = -.067, *p* < .474	0.45
Electronic	*r*(114) = .495, *p* < .001	24.50	*r*(113) = .204, *p* < .029	4.16

*PC* partial correlation

## Discussion and conclusion

The aim of this study was to investigate music-colour synaesthesia from a novel perspective of grounding in action. Complementing previous views, special emphasis was placed on bodily action and its experience, starting from the hypothesis that music-colour synaesthesia may share similar action-based properties with more typical music cognition (Curwen, 2020). We compared the contribution of the mediating factor of emotional responses to music to synaesthesia with the mediating factor of sensorimotor associations, and the effect of performing music on their principal, an unknown or an electronic, dead-pan instrument.

An important contribution of the study is the clarification of the relevance of sensorimotor associations with music to music-colour synaesthesia above and beyond the relevance of emotional associations. Whilst emotions may play a role as mediating factor (Isbilen & Krumhansl, 2016; Palmer et al., 2013, 2016), the role of sensorimotor associations need to be considered in conjunction as they may in turn be a mediating factor in emotional connotations with music (Eitan et al., 2018; Irrgang & Egermann, 2016; Molnar-Szakacs & Overy, 2006). This is in line with Clarke (2014) who explores the integration of perception, emotion, and embodiment in the experience of music emphasising the significance of phenomenological qualities and the dynamic aspects of human subjectivity. This perspective goes beyond the emotion-cognition divide and emphasises the reciprocal relationship between perception and action.

The close interrelationship and their partial independence were confirmed in the PCA analyses of the dimensions, which showed three mixed components and one sensorimotor component. The inclusion of both emotional mediation and sensorimotor associations in this study nuances the conventional notion of a direct, unmediated mapping between senses (from sense-to-sense). This perspective offers a departure from traditional experiments and models, which primarily focus on testing direct links between specific musical properties, such as pitch height, and the perception of colour (Gallace & Spence, 2006; Marks, 1987, 2004; Mondloch & Maurer, 2004).

Moreover, as far as we are aware, this is the first experimental study investigating the hypothesis of music-colour synaesthesia being grounded in human action. On this basis, we predicted that the lived experience of playing a musical instrument could be a significant factor in influencing the intensity of induced synaesthesia. This hypothesis was not confirmed (H1). We did confirm a significant influence on the strength of synaesthesia of whether the music was performed by a human or artificially generated (H2). The other hypotheses were also confirmed of parallels in emotional and sensorimotor processing of music in Controls and Synaesthetes (H3), connections between emotion ratings and synaesthesia (H4), but a stronger relationship between sensorimotor responses and synaesthesia (H5). In combination, we take this as evidence for the hypothesis that changes to action-related qualities of a musical stimulus affect the resulting synaesthetic experience in some forms of music-colour synaesthesia.

The lack of an effect of familiarity with an instrument could be due to limitations in sample size of the study, limited instrumental experience of some of the participants, and due to variability in forms and manifestations of synaesthesia among participants. The identification of genuine synaesthetes remains difficult and is well documented in synaesthesia research as being complex (Eagleman et al., 2007; Rothen et al., 2013). For the purpose of this study, music-colour synaesthesia was described to participants as “spontaneously seeing colours and shapes on hearing music”. All synaesthetes were self-identifying via an online survey and no formal verification methods were used. However, all potential synaesthetes were asked to provide an example of a piece of music that elicits a very strong synaesthetic experience and one that does not, and a brief description of why (see Appendix C). Phase 2 of the survey also invited synaesthetes to describe their experience as well as to rate it, and most offered rich and detailed explanations to support their synaesthesia (see Appendix D). The open-ended responses confirmed in our reading the self-report of synaesthesia, but its presence was not objectively verified. Typically occurring in only 4% of the general population (Gallese, 2005, 2014) its rarity in occurrence challenges recruitment of large numbers of synaesthetes. A sample size of 29 is comparatively large in synaesthesia research, comparable to studies on cross-modal associations (Auvray and Deroy, 2015; Overy & Molnar-Szakacs, 2009; Spence, 2011; Timmers & Li, 2016) and within requirements to test within-participants effects of a moderate effect size (Cohen, 1988).

It is also established that specific manifestations of synaesthesia vary from person to person, a reason for some research to focus on single cases (Mills et al., 2003). Variability across participants and depending on musical materials meant that we examined the relative strength of responses across a number of dimensions irrespective of the specific dimensions that were activated. Future research may examine these specific associations in more detail and fine-tune the choice of dimensions in relation to the materials employed. The lack of an effect of familiarity with an instrument in our findings, therefore, does not mean that a synaesthetic response that is specific to a musician’s musical instrument does not exist. Indeed, we would argue that many forms of music-colour synaesthesia are still to be discovered. Future research specifically interested in this hypothesis may want to use this form of synaesthesia as an inclusion criteria and test synaesthetic responses using experimental methods designed to test occurrence of synaesthesia e.g., as in Curwen (2022a) and Ward et al., (2006b).

In conclusion, this research places synaesthesia and typical music cognition on a continuum as both being grounded in action. This complements previous views that hold that they rely on distinct neurocognitive mechanisms, and supports findings of parallel results between controls and synaesthetes (e.g., Isbilen & Krumhansl, 2016). Similarities between synaesthetes and non-synaesthetes suggest that synaesthesia may be a development of typical cross-modal sensorimotor associations. However, the automaticity, consistency, and specificity of inducer and congruent pairings, along with its manifestation as a conscious percept-like experience, differentiate synaesthetes from non-synaesthetes.

By offering empirical evidence for a theoretical argument previously published (Curwen, 2020) this research has several important implications for future research that should take into account the role of sensorimotor associations (in addition to emotion) including related to the performance of music, when considering synaesthesia. This concerns synaesthetic responses to music in musicians, but the arguments and supporting findings of music cognition as grounded in action (Krueger, 2009, 2011, 2014; Loaiza, 2016; Maes et al., 2014; Reybrouck, 2014, 2017; Schiavio et al., 2017, 2019) suggest that this is likely to generalise to populations without extensive musical performance experience.

### Supplementary Information

Below is the link to the electronic supplementary material.

Supplementary file1 (DOCX 22 KB)

## Data Availability

The data used in the analysis may be found at https://doi.org/10.15131/shef.data.16943941.v1 (Curwen, 2022b).

## Footnotes

1 “Inducer”—a percept or concept (such as sound, or an idea**)** that triggers an experience in another modality (such as colour or texture) (Grossenbacher & Lovelace, 2001).

2 The movements of a Baroque Suite are also known as dances.
