# Supplementary material for: Action, emotion, and music-colour synaesthesia: an examination of sensorimotor and emotional responses in synaesthetes and non-synaesthetes
Source: Psychol Res. 2023 Jul 15;88(2):348–62. doi: 10.1007/s00426-023-01856-2 (PMC10857979; doi:10.1007/s00426-023-01856-2)
Supplement: Supplementary file 1 — Supplementary file1 (DOCX 22 KB) [file 426_2023_1856_MOESM1_ESM.docx]

APPENDICES

Appendix A – Questions in Phase One of the SmartSurvey Form

**All data will be anonymised, but so that an identification number can be assigned to your responses please provide the following:**

Last 2 letters of your surname

Month of you birth, i.e., 05

First letter of the town where you were born

Please provide your email address.

What is your age?

What is your nationality?

What is your gender?

**Please rate the level of expertise you have acquired on your principal instrument.**

Beginner Intermediate Grade 8 or equivalent Very high level amateur Professional

**Do you have perfect pitch?**

Yes

No

Don’t know

**Please choose your principal instrument**

Cello Bassoon Other (please specify):

Violin Oboe

Double Bass Piano

Guitar Marimba

Clarinet Trumpet

Bass Clarinet Trombone

Flute French horn

Saxophone Viola

**Do you enjoy listening to electronic music?**

I have no experience with electronic music

No

Some types

Yes

**Please play this excerpt and select one of the following options that best describes your response to it:**

I love this performance on the Marimba

I am OK with listening to Bach being performed on the Marimba

I cannot stand listening to Bach being performed on the Marimba or any instrument other than the one it was originally written for

**Do you ever experience colours on hearing music?**

Yes

No

**Please indicate all the stimuli that might lead you to experience colours on hearing music:**

Musical keys - sounded

Musical keys - association with (not necessarily sounded)

Musical tones

Different instruments/timbre

Compositional style

Different composers

Musical notation

Other (please specify):

**How would you describe your experience of synaesthetic colour?**

Being in my mind's eye, or of 'knowing' the colour

Projected outside the body into external space

**Please provide an example of a piece of music that elicits a very strong synaesthetic experience and one that does not, and a brief description of why.**

**Do you experience any other form of synaesthesia?**

Yes

No

**Select any of the following that are experiences you are familiar with:**

Colours for letters and/or numbers

Colours for days of the week or months

Auditory-tactile synaesthesia - certain sounds induce sensations in parts of the body

Spatial sequence synaesthesia - numerical sequences seen as points in space

Mirror-touch synaesthesia - feeling same sensation as other persons feels

Lexical-Gustatory Synaesthesia - different kinds of tastes when hear certain words or phonemes

Other (please specify):

**So that you can be correctly directed to Part Two of the study, please select the pairing of instruments that includes your principal instrument. If you have played the second instrument before, please enter an alternative instrument from the list that you have never played in the 'Other' box.**

Cello and Bass clarinet

Violin and Flute

Viola and Bassoon

Double Bass and Trumpet

French Horn and Clarinet

Oboe and Trombone

Saxophone and Marimba

Piano and Guitar

Other (please specify)

# Appendix B – Instrumental Pairings Selected by Participants

| Instrumental Pairing | Synaesthetes | Controls |
| --- | --- | --- |
| Guitar & Piano | 14 | 7 |
| Violin & Flute | 1 | 4 |
| Oboe & Trombone | - | 2 |
| Cello & Bass Clarinet | 2 | 1 |
| Sax & Marimba | 1 | 2 |
| Trumpet & Double Bass | - | 3 |
| Viola & Bassoon | 1 | 1 |
| Clarinet & Piano | 2 | - |
| Bassoon & Piano | 1 | 1 |
| Sax & Piano | 2 | - |
| Bass Guitar & Clarinet | - | 1 |
| Bassoon & Guitar | 1 | - |
| Clarinet & French Horn | 1 | - |
| Flute & Viola | - | 1 |
| Guitar & Trumpet | - | 1 |
| Guitar & Clarinet | - | 1 |
| Guitar & Flute | - | 1 |
| Guitar & Trombone | - | 1 |
| Guitar & Violin | 1 | 1 |
| Harp & Clarinet | - | 1 |
| Harp & Trumpet | - | 1 |
| Harp & Bassoon | - | 1 |
| Piano & Viola | 1 | - |
| Piano & Cello | 1 | - |
| Recorder & Oboe | - | 1 |
|  |  |  |

#

# Appendix C – List of Performances Used to Create Stimuli

| Instrument | Artist | Link to Source |
| --- | --- | --- |
| Vc | Mischa Maisky | <https://tinyurl.com/2mc8epxy> |
| Vl | Rachel Podger | <https://tinyurl.com/3nz3sych> |
| Sax | Andrew Richard Dahlke | <https://tinyurl.com/2p9frwkh> |
| Va | Lillian Fuchs | <https://tinyurl.com/2p87pbjb> |
| Db | Jeff Bradetich | <https://www.youtube.com/channel/UCdFEFZ6HZ5ZD_SibGDAJlGg> |
| Cl | Daniel Silver | <https://www.youtube.com/watch?v=3DIIlr19VMw&t=11s> |
| Cl | Peter Koetsveld | <https://tinyurl.com/24nvud82> |
| Cl | Wilfried Berk | <https://youtu.be/CVkKiJ_D8LQ> |
| Pn | Eleanor Bindman | <https://tinyurl.com/3stpyf8h> |
| Ob | Gonzalo X Ruiz | <https://tinyurl.com/bp9eekz6> |
| Bcl | James T Shields | <https://youtu.be/nmA1biVyow4> <https://youtu.be/dSI9xmR2gOY> |
| Fl | Sabine Kittle | <https://tinyurl.com/3d6npeha> |
| Mar | Jean Geoffrey | <https://tinyurl.com/ycknj43j> |
| Bn | William Ludwig | <https://tinyurl.com/2p93wrmr> |
| Trpt | David Cooper | <https://tinyurl.com/bdh7szm4> |
| Hn | Jacek Muzyk | <https://tinyurl.com/2p888brt> |
| Gui | Jeffrey McFadden | <https://tinyurl.com/4wt5hbhh> |
| Tbn | Denson Paul Pollard | <https://tinyurl.com/3vrzamhu> |
| Hp | Victoria Drake | <https://tinyurl.com/yyy22sue> |
| Rec | Bolette Roed | <https://tinyurl.com/mur2pha8> |
| Bgtr | Ariane Cap | <https://tinyurl.com/mr4636vu> |
| *Note.* Pn = Piano, Gui = Guitar, Mar = Marimba, Sax = Saxophone, Vc = Cello, Bn = Bassoon, Cl = Clarinet, Vn = Violin, Fl = Flute, Bcl = Bass Clarinet, Va = Viola, Hn = French Horn, Hp = Harp, Tpt = Trumpet, Bgtr = Bass Guitar, Tbn = Trombone, Rec = Recorder, Db = Double Bass, Ob = Oboe | | |

# Appendix D – Questions in Phase Two of the SmartSurvey Form

**Please play the above excerpt and answer the following questions:

Please indicate the strength of your motivation to move and vocalise to the music.**

|  | Not at all | Weakly | Moderately | Strongly | Very Strongly |
| --- | --- | --- | --- | --- | --- |
| Imagined sing/hum along? |  |  |  |  |  |
| Imagined move/dance along? |  |  |  |  |  |
| Actually move/dance along? |  |  |  |  |  |
| Actually sing/hum along? |  |  |  |  |  |

**Please rate the applicability and intensity of the following terms (Left column A and Right column B) that best describe your listening experience.**

|  | Very Strong A | Strong  A | Moderate A | Weak  A | N/A | Weak  B | Moderate B | Strong  B | Very Strong B |  |
| --- | --- | --- | --- | --- | --- | --- | --- | --- | --- | --- |
| Spiky |  |  |  |  |  |  |  |  |  | Round |
| Happy |  |  |  |  |  |  |  |  |  | Sad |
| Positive |  |  |  |  |  |  |  |  |  | Negative |
| Pleasant |  |  |  |  |  |  |  |  |  | Unpleasant |
| Spatially High |  |  |  |  |  |  |  |  |  | Spatially Low |
| Rough |  |  |  |  |  |  |  |  |  | Smooth |
| Thick |  |  |  |  |  |  |  |  |  | Thin |
| Excited |  |  |  |  |  |  |  |  |  | Calm |
| Bright |  |  |  |  |  |  |  |  |  | Dark |
| Dynamic |  |  |  |  |  |  |  |  |  | Static |
| Light |  |  |  |  |  |  |  |  |  | Heavy |
| Tense |  |  |  |  |  |  |  |  |  | Relaxed |
| Weak |  |  |  |  |  |  |  |  |  | Strong |
| Angry |  |  |  |  |  |  |  |  |  | Tender |
| Warm |  |  |  |  |  |  |  |  |  | Cold |

**Please rate the extent and the intensity of your synaesthetic experience:**

|  | Not at all | Weakly | Moderately | Strongly | Very Strongly |  |
| --- | --- | --- | --- | --- | --- | --- |
| Dark/light shades |  |  |  |  |  |  |
| Movement |  |  |  |  |  |  |
| Shapes |  |  |  |  |  |  |
| Colours |  |  |  |  |  |  |
| Textures |  |  |  |  |  |  |

Please detail any other experience:

#

# Appendix E – Example of synaesthetic experience from self-reporting synaesthetes

“Tamino - Arte concert (Les Concerts Volants), only audio. I don't know exactly why specific pieces elicit strong synaesthetic experience and why not, but I guess it has something to do with the complexity, "movement", melodies and harmonies in the piece of music. I think I see melodies as forms and harmonies as a basic layer of colours and texture. In this concert I see strong colours and texture but not overloaded, I feel tones over my skin, in my stomach, in my nose, I have the impression I hear the sound in front of my face in canvas, not through my ears. Listening to classical music is even stronger, but sometimes I cannot bear it because it is overwhelming, so I don't listen to it too much. I cannot even walk with earphones when listening to classical music. Also, but in a nice way, ‘Max Richter: The four seasons recomposed. Spring. I have no synaesthetic experience when listening to metal music or techno without melodies of harmonies. It hurts my ears, makes me very uncomfortable, that's why I don't listen to it.”

‘Synaesthetic experience: Isolde's Liebestod by Wagner / No synaesthetic experience: a typical Mozart sonata. Some music elicits a 'feeling' of colour in my mind's eye (I don't actually see the colours). It's mostly music with interesting harmonies and a lot of harmonic changes. I don't get a synaesthetic experience every time: it depends on my mood, level of attention spent to the music and the level of emotional engagement to the music. I suppose I do not experience anything with a typical Mozart sonata because this music is generally very transparent and balanced with respect to harmony.’
